# The afterload-dependent peak efficiency of the isolated working rat heart is unaffected by streptozotocin-induced diabetes

**DOI:** 10.1186/1475-2840-13-4

**Published:** 2014-01-05

**Authors:** June-Chiew Han, Soyeon Goo, Carolyn J Barrett, Kimberley M Mellor, Andrew J Taberner, Denis S Loiselle

**Affiliations:** 1 Auckland Bioengineering Institute, The University of Auckland, Auckland, New Zealand; 2 Department of Physiology, The University of Auckland, Auckland, New Zealand; 3 Department of Engineering Science, The University of Auckland, Auckland, New Zealand

**Keywords:** STZ-induced diabetes, Diabetic heart, Cardiac work, Cardiac oxygen consumption, Cardiac efficiency, Efficiency-afterload relation

## Abstract

**Background:**

Diabetes is known to alter the energy metabolism of the heart. Thus, it may be expected to affect the efficiency of contraction (i.e., the ratio of mechanical work output to metabolic energy input). The literature on the subject is conflicting. The majority of studies have reported a reduction of myocardial efficiency of the diabetic heart, yet a number of studies have returned a null effect. We propose that these discrepant findings can be reconciled by examining the dependence of myocardial efficiency on afterload.

**Methods:**

We performed experiments on streptozotocin (STZ)-induced diabetic rats (7-8 weeks post-induction), subjecting their (isolated) hearts to a wide range of afterloads (40 mmHg to maximal, where aortic flow approached zero). We measured work output and oxygen consumption, and their suitably scaled ratio (i.e., myocardial efficiency).

**Results:**

We found that myocardial efficiency is a complex function of afterload: its value peaks in the mid-range and decreases on either side. Diabetes reduced the maximal afterload to which the hearts could pump (105 mmHg versus 150 mmHg). Thus, at high afterloads (for example, 90 mmHg), the efficiency of the STZ heart was lower than that of the healthy heart (10.4% versus 14.5%) due to its decreased work output. Diabetes also reduced the afterload at which peak efficiency occurred (optimal afterload: 63 mmHg versus 83 mmHg). Despite these negative effects, the *peak* value of myocardial efficiency (14.7%) was unaffected by diabetes.

**Conclusions:**

Diabetes reduces the ability of the heart to pump at high afterloads and, consequently, reduces the afterload at which peak efficiency occurs. However, the peak efficiency of the isolated working rat heart remains unaffected by STZ-induced diabetes.

## Background

**Figure 1:**
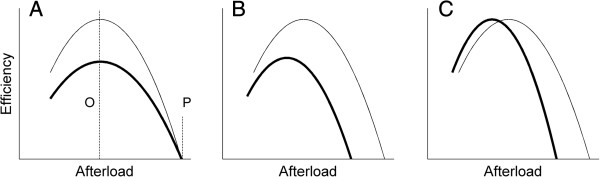
**Schematic representations of three possible relations between myocardial efficiency and left-ventricular afterload.** Myocardial efficiency as a function of afterload proposed for the control heart (thin line) and for the diabetic heart (thick line). The diabetic heart could have **(A)** a lower value of peak efficiency (which occurs at the optimal afterload as indicated by the broken line labelled ‘O’) but can pump to the same peak afterload (the broken line labelled ‘P’), lower peak efficiency and lower peak afterload **(B)**, or same peak efficiency but lower peak afterload **(C)**.

Cardiac efficiency (calculated from the ratio of work to oxygen consumption) has been used extensively as an index of energetics of the myocardium. In many studies of the energetics of the diabetic heart, the deterioration of cardiac performance has been linked to a reduction of cardiac efficiency [1-5]. But, some studies have reported an unchanged value in diabetic animals [6-9]. We propose that these conflicting findings are a consequence of reporting only a single value of efficiency. As experimentally shown using one-dimensional cardiac preparations, such as papillary muscles [10-16] and trabeculae carneae [17,18], cardiac efficiency is a complex function of afterload (Figure 1). It has a peak value somewhere in the middle of the afterload range. Hence, reporting only a single value of efficiency is somewhat incomplete, and could perhaps mislead the author into reaching a faulty conclusion, even to the extent of erring on the *direction* of the difference. We elaborate this point using Figure 1, where we propose three cases.

We term the afterload at which the efficiency peaks the ‘optimal afterload’, as distinct from ‘peak afterload’ where work (and, consequently, efficiency) is zero. For the first case (Figure 1A), compared with the control heart, the diabetic heart has a lower value of peak efficiency, but the same optimal afterload and the same peak afterload. Adoption of the same peak afterload is based on experimental results showing no difference in the left-ventricular systolic pressure [1,2,19,20]. But, there exists evidence that the left-ventricular systolic pressure is lower in the diabetic heart [4,9,21-25], which leads us to consider two other cases, in which the diabetic heart has a lower peak afterload and a lower optimal afterload, but its peak efficiency can be either lower than (the second case; Figure 1B), or the same as (the third case; Figure 1C), that of the control heart. In all three cases, one would reach the conclusion that the diabetic heart has a lower cardiac efficiency if the experiment had been performed at a high afterload. But, if measurements had been made at or around the optimal afterload (the third case), then one would find that the diabetic heart has the same efficiency as the control heart.

The objectives of our study were hence threefold: (i) to determine the effect of diabetes on cardiac mechano-energetics, (ii) to extend the single value of cardiac efficiency of the diabetic heart, as reported in the literature, to its expression as a function of afterload, and (iii) to reconcile the conflicting findings showing either a decrease, or no change, of the cardiac efficiency of the diabetic heart. We conducted experiments using streptozotocin-induced diabetic hearts isolated from rats. Each heart, under working mode, was challenged to a series of afterloads from 40 mmHg to its maximal capacity.

## Methods

### Animal preparation

Male Sprague–Dawley rats (6-7 weeks old, 250-300 g) were made diabetic by a single tail vein injection of streptozotocin (STZ; 55 mg/kg). Control (SHAM) rats were given an intravenous injection of an equivalent volume of saline. Rats in each group were housed in pairs in a cage at room temperature (20-22°C) on a 12-hr light/dark cycle with *ad libitum* access to food (standard rat chow) and tap water. Blood glucose level was measured daily for the first week and once per week thereafter for 7-8 weeks. Induction of diabetes in the STZ-treated rats was confirmed by blood glucose >20 mM. Experiments were conducted in accordance with protocols approved by The University of Auckland Animal Ethics Committee.

### Measurement of blood pressure

From each group, 4 rats were subjected to measurement of blood pressure *in vivo*. During the surgical period, they were continuously anesthetised using inhaled isoflurane (2.5%). Under sterile conditions, each rat was surgically implanted with a blood pressure telemeter (Model TRM53P, Telemetry Research, Auckland, NZ) with a solid-state pressure sensor at the tip of the catheter. The abdominal aorta was cannulated 1–2 cm rostral to the iliac bifurcation and the sensor tip of the telemeter advanced proximally 1–2 cm. Pain relief/analgesia (Temgesic, 20 mg/kg) and antibiotic (Baytril 2.5% solution, 0.8 mL/kg) were administered at the time of the surgery. The rats were monitored closely and analgesia given daily for up to 3 days after the surgery.

The arterial pressure signal was sampled at 500 Hz using an analogue–digital data acquisition card (PCI 6024E, National Instruments, TX, USA) and continuously displayed by a data acquisition programme (Universal Acquisition 11, University of Auckland, Auckland, NZ). Heart rate, and systolic and diastolic blood pressures were derived from the arterial pressure waveform. For the calculation of the overall average level of mean arterial blood pressure, the sampled signals were averaged every 2 s. All subsequent data collection and analyses were performed using the same data analysis programme. Rats were monitored for at least 12 days to ensure they were well-recovered from surgery, with the data presented being the mean values for the last 5 days of the recordings (7-8 weeks post-injection).

### Heart preparation

At 7-8 weeks post-injection, a rat was deeply anaesthetised with isoflurane (5% in O_2_), injected with heparin (1000 IU/kg), and body mass measured. Following cervical dislocation, the heart and lungs were excised and plunged into chilled Tyrode solution. The aorta was immediately cannulated for Langendorff perfusion (afterload 70 mmHg) with Tyrode solution at room temperature. The lungs were then separated from the heart. The Tyrode solution had compositions of (in mM): 130 NaCl, 6 KCl, 1 MgCl_2_, 0.5 NaH_2_PO_4_, 1.5 CaCl_2_, 10 Hepes, 10 glucose, its pH adjusted to 7.4 using Tris and was vigorously bubbled with 100% O_2_. Glucose at a concentration of 10 mM has been shown to be sufficient to optimise the mechanical performance of the diabetic heart [26].

The venae cavae (right superior, left superior and inferior) were ligated, as were three of the pulmonary veins, leaving one for cannulation. The pulmonary artery was also cannulated. Oxygen sensors (FOXY-R-8CM, NeoFox^@^ Phase Measurement System, Ocean Optics Inc., FL, USA), with their associated temperature probes, were inserted into the aortic cannula (to reside just above the coronary ostia) and the arterial cannula, for measuring the coronary arterial partial pressure of O_2_ (PO_2_) and the coronary venous PO_2_, respectively. Flow probes, connected to flow meters (T206 and T106, Transonic System Inc., NY, USA), measured the rate of aortic outflow and the rate of coronary outflow. The fully-instrumented heart was enveloped by a water-jacketed glass chamber, and the temperature elevated to 32°C. Finally, the intrinsic heart rate was measured and a unipolar stimulus electrode (Coaxial Stimulation Electrode, Harvard Apparatus, MA, USA) was placed on the surface of the right atrium and the heart was paced at 4 Hz (which, in all cases, was above the intrinsic rate of beating).

### Experimental protocol

Upon attaining steady-state of the rate of coronary flow under Langendorff mode, perfusion was then switched to working-heart mode (preload 20 mmHg and afterload 70 mmHg). Preload and afterload (measured using in-line physiological pressure transducers: SP 844 Transducer, MEMSCAP Inc., NC, USA) were varied by changing the height, above the mid-level of the heart, of the preload chamber and the aortic outflow tubing, respectively. The afterload for the RV was fixed at 10 mmHg. When each of the measured variables had reached steady states, the afterload was altered to a new value. Afterloads were presented in random order within the range between 40 mmHg and maximal (at which point aortic flow was near zero). When the series of afterload interventions was completed, perfusion was switched back to Langendorff mode (afterload 70 mmHg) to measure the basal rate of VO_2_ of the heart (cardiac arrest induced by high KCl, 26 mM). Data were continuously acquired and recorded using PowerLab LabChart Pro. software (ADInstruments, Dunedin, NZ). In total, 16 STZ-diabetic hearts and 17 SHAM hearts were used in this study.

### Trans-epicardial exchange of oxygen

Myocardial VO_2_ was corrected for the trans-epicardial exchange of oxygen with the surrounding air in the glass chamber housing the heart. During the course of an experiment, the gaseous content in the glass chamber was flushed with 21% O_2_. Upon completion of the afterload series, the gaseous content in the glass chamber was changed to 100% O_2_ and the afterload was set at 50 mmHg. This resulted in an increase in the measured venous PO_2_ and, consequently, a decrease in the calculated steady-state rate of myocardial oxygen consumption, reflecting a diminution in the loss of oxygen from the coronary circulation across the epicardial surface. The true value of myocardial VO_2_ was calculated by making use of the values measured in the presence of 21% and 100% O_2_ in the heart chamber, as previously described [27,28].

### Calculations

The rate of performance of work ($\dot{W}$) was given by the product of the afterload and the total (coronary plus aortic) flow. The rate of oxygen consumption was given by the product of the arterio-venous difference in PO_2_ and the coronary flow, assuming an oxygen solubility of 0.0249 mL atm^-1^ mL^-1^ at 32°C [27], and the enthalpy equivalent of O_2_ to be 20 J mL^-1^[29]. ‘Total efficiency’ (*ε*_*Total*_) was defined as the ratio of the rate of performing work to the rate of total enthalpy production $\left(\Delta {\dot{H}}_{\mathrm{Total}}\right)$. ‘Mechanical efficiency’ (*ε*_*Mech*_) was calculated from total efficiency by subtracting the rate of enthalpy production in the basal state $\left(\Delta {\dot{H}}_{\mathrm{Basal}}\right)$. Explicitly:

(1) $$\epsilon_{\mathrm{Total}}=\frac{\dot{W}}{\Delta {\dot{H}}_{\mathrm{Total}}}$$

(2) $$\epsilon_{\mathrm{Mech}}=\frac{\dot{W}}{\Delta {\dot{H}}_{\mathrm{Total}}-\Delta {\dot{H}}_{\mathrm{Basal}}}$$

### Normalisation of data

At the end of each experiment, the ventricles were opened to measure the thickness of the septum and the LV and RV walls. The heart was then blotted and its wet mass measured. Work and VO_2_ were expressed per beat by dividing by stimulus frequency (4 Hz). The work, total *VO*_*2*_, aortic and coronary flow rates, and basal rate of *VO*_*2*_ were normalised to heart wet mass and thus expressed in units of J kg^-1^, mL min^-1^ kg^-1^ or W kg^-1^, where appropriate. Whereas the tissues of both ventricles are perfused (and actively contracting), the RV performs work against a negligible afterload (10 mmHg). As a result, by normalising oxygen consumption to the mass of the entire heart, we slightly underestimate the work (and, hence, efficiency) of the whole-heart but have no reason to expect any difference between hearts of STZ and SHAM animals.

### Data fitting

Aortic flow, coronary flow and VO_2_, were plotted against afterload and the data fitted using 3^rd^-order polynomials. Work and total *ε*_*Total*_ were likewise plotted against afterload and fitted using 3^rd^-order polynomials but with intercepts set to zero (since work is zero at zero afterload). The regression lines within a group (16 for the STZ group and 17 for the SHAM group) were averaged using the Nonlinear Mixed Model (PROC NLMIXED) of the SAS software package. Regression coefficients were assumed to have arisen from a multivariate normal probability distribution [30].

### Statistical analyses

Parameters of interest, estimated from each regression line defining the dependence of the measured variables on afterload (i.e., peak value, optimal afterload and peak afterload), were averaged and expressed as means ± standard error (SE). The data arising from all of the dependent variables were analysed using Hotelling’s T^2^, the two-group (STZ versus SHAM) equivalent of MANOVA (Multivariate Analysis of Variance), prior to performing multiple ANOVAs on individual variables. Means were adjudged to be different if *P* < 0.05. Note that, for a 5% risk of a Type I Error, and with *n* = 16 or 17 observations per group, the statistical power to detect a separation of means of at least one standard deviation is 80% [31].

## Results

### Morphometric characteristics of the rats

As displayed in Table 1, compared with the control rats (SHAM), the streptozotocin (STZ)-induced diabetic rats had elevated blood glucose. They had, on average, smaller body mass, shorter tibial length and smaller hearts. Their average heart wall thickness did not differ for either the left ventricle (LV) or the septum, but was higher in the right ventricle (RV) of the STZ group. When the wall thickness was expressed as a fraction of heart wet mass, the difference in mean relative RV thickness was no longer statistically significant, but the mean relative thickness of both the septum and LV were greater. No statistical difference was detected for the average lung wet mass, but when expressed as a percentage of body mass, the STZ rats had a greater mean relative lung mass.

**Table 1:** General features of SHAM and STZ rats

**Parameter**	**SHAM**	**STZ**
Body mass (g)	538 ± 15	375 ± 13*
Tibial length (mm)	45.4 ± 0.6	42.2 ± 0.6*
Lung mass (g)	2.05 ± 0.09	1.89 ± 0.06
Lung mass/body mass (%)	0.38 ± 0.02	0.52 ± 0.03*
Heart mass (g)	1.49 ± 0.05	1.16 ± 0.04*
Heart mass/body mass (%)	0.28 ± 0.01	0.31 ± 0.01*
RV wall thickness (mm)	1.33 ± 0.04	1.16 ± 0.06*
LV wall thickness (mm)	3.80 ± 0.09	3.74 ± 0.09
Septal wall thickness (mm)	3.18 ± 0.07	3.14 ± 0.11
RV thickness/heart mass (mm g^-1^)	0.91 ± 0.03	1.02 ± 0.06
LV thickness/heart mass (mm g^-1^)	2.60 ± 0.10	3.26 ± 0.12*
Septal thickness/heart mass (mm g^-1^)	2.16 ± 0.07	2.74 ± 0.13*
Blood glucose (mM)	6.8 ± 0.1	29.6 ± 0.5*
Systolic blood pressure (mmHg)	129 ± 2.8	125 ± 2.9
Diastolic blood pressure (mmHg)	84 ± 2.2	82 ± 2.2
Mean arterial blood pressure (mmHg)	103 ± 2.3	102 ± 2.6
Heart rate *in vivo* (beats min^-1^)	346 ± 16	302 ± 5*
Heart rate *in vitro* (beats min^-1^)	175 ± 7	156 ± 5*

Values are means ± SE. In all cases, *n* = 17 for SHAM and *n* = 16 for STZ, except for blood pressure and heart rate *in vivo,* both *n* = 4.

Asterisks indicate statistically significant differences in mean values between SHAM and STZ: *P < 0.05.

The average *in vivo* levels of systolic, diastolic, and mean arterial blood pressures did not differ between STZ and control groups, but the average heart rate of the former was lower, both *in vivo* and *in vitro* (i.e., at the experimental temperature of 32°C).

### Coronary PO_2_ as a function of afterload

**Figure 2:**
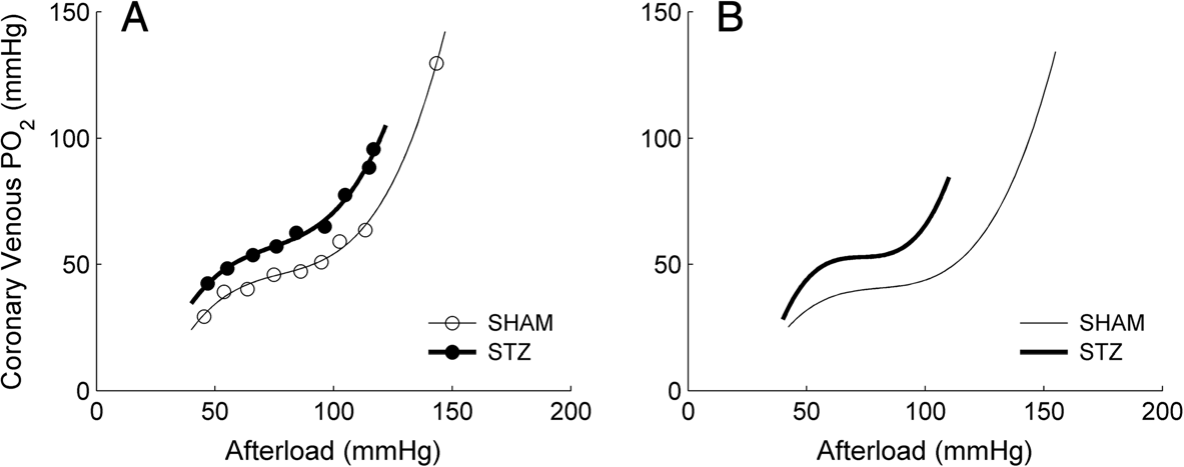
**Coronary venous PO**_**2 **_**as a function of afterload. A**: representative data from a STZ (filled symbols) and a SHAM (open symbols) working heart. **B**: average relations. Each line (thick lines: STZ; thin lines: SHAM) represents the average of the *n* = 16 lines (each from a STZ heart) or *n* = 17 lines (each from a SHAM heart).

Panels A and B of Figure 2 show, respectively, the relation between coronary venous partial pressure of oxygen (PO_2_) and afterload for individual hearts (STZ and SHAM) and for the average measurements recorded from all hearts. Note the progressive *increase* of venous PO_2_ with metabolic energy expenditure, a result consistently observed in every heart. Diabetes did not alter the profile of the venous PO_2_-afterload relation. There was no statistical difference between the two groups in the mean values of coronary venous PO_2_, particularly at the specific pressures of interest: 70 mmHg and 90 mmHg (see below). Likewise, coronary arterial PO_2_ was constant (690 mmHg) with afterload for both STZ and SHAM cohorts (data not shown).

### Aortic and coronary flow rates as functions of afterload

**Figure 3:**
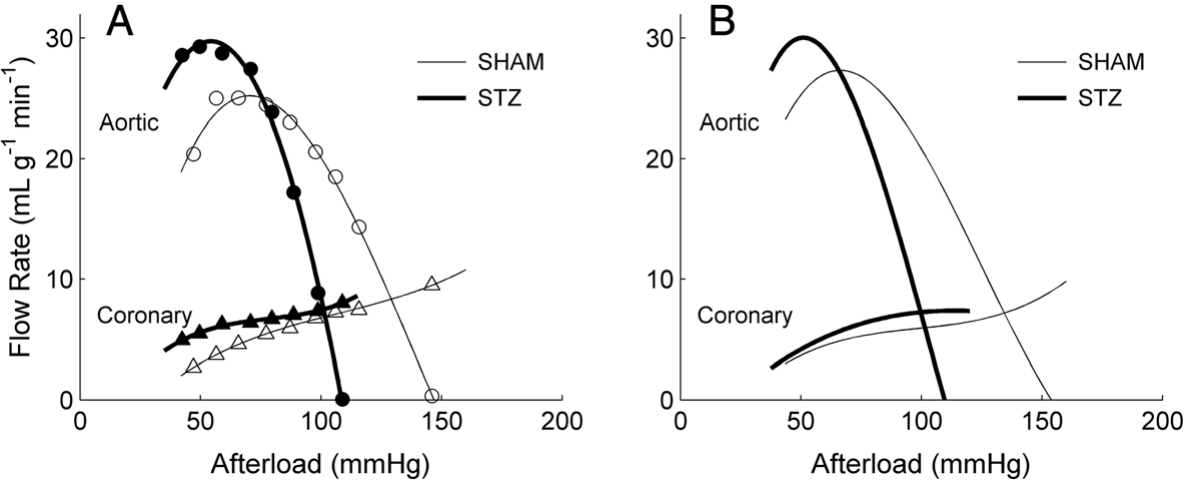
**Aortic and coronary flow rates as functions of afterload.A**: representative data (circles: aortic flow rates, triangles: coronary flow rates) from a STZ (filled symbols) and a SHAM (open symbols) working heart. **B**: average relations. Each line (thick lines: STZ; thin lines: SHAM) represents the average of the *n* = 16 lines (each from a STZ heart) or *n* = 17 lines (each from a SHAM heart). The variability of the relations in B is quantified in Tables 2 and 3.

Each heart was electrically stimulated at 4 Hz to avoid the confounding effects of difference in intrinsic heart rates (i.e., STZ: 2.6 ± 0.1 Hz and SHAM: 2.9 ± 0.1 Hz; Table 1). Under working mode, and at various afterloads, both aortic and coronary flow rates from each heart were measured and were plotted as functions of afterload (Figure 3A: representative data; Figure 3B: average relations). The afterload series was terminated when the heart neared its limit to pump, i.e., when aortic flow rate was in the vicinity of zero. Note that the STZ hearts were unable to pump to as high an afterload as the SHAM hearts (105 ± 2 mmHg versus 150 ± 4 mmHg).

### Work, total oxygen consumption and total efficiency as functions of afterload

**Figure 4:**
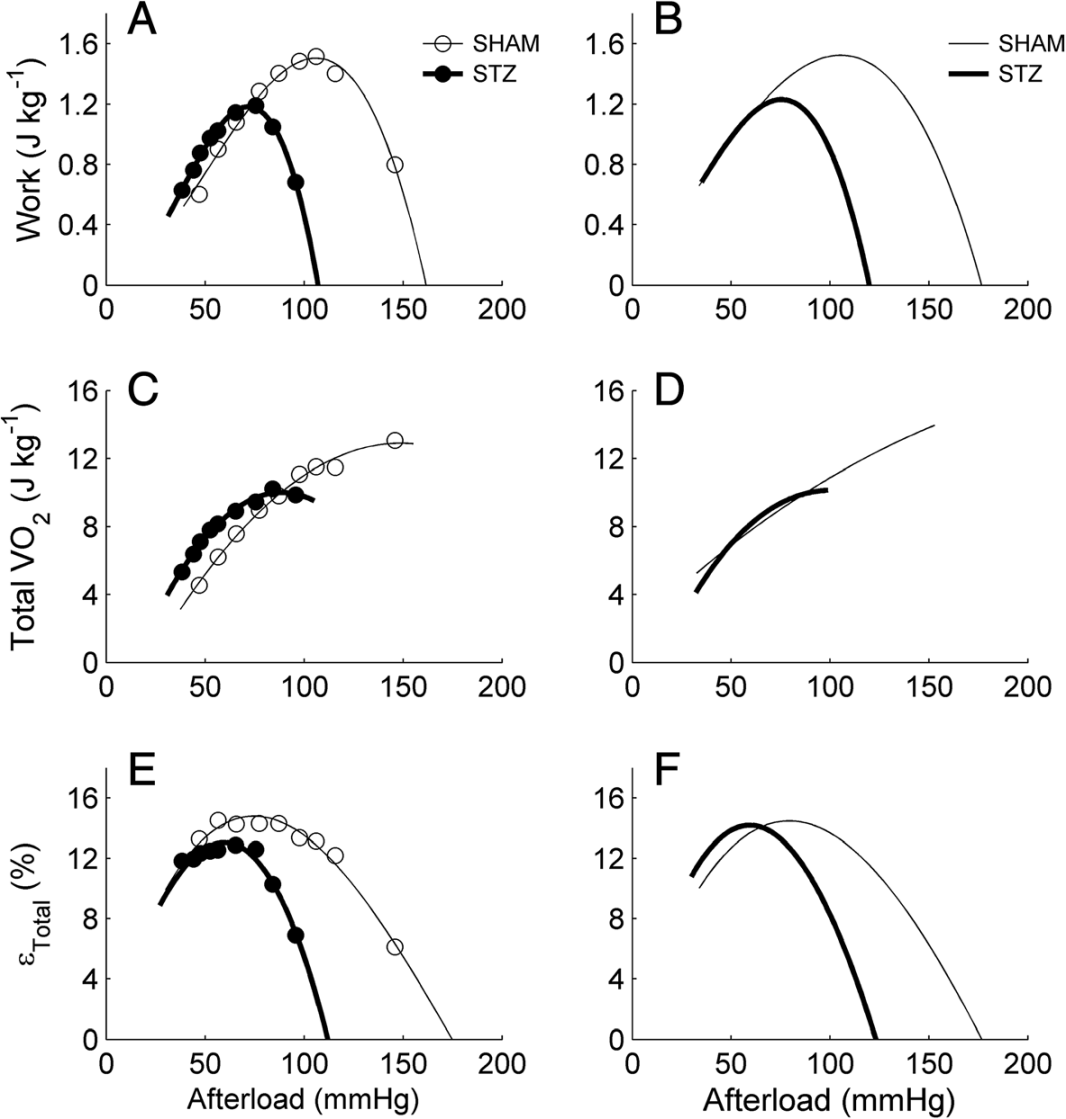
**Mechano-energetics variables as functions of afterload.** Work **(A and B)**, total oxygen consumption (VO_2_; **C** and **D**) and total efficiency (*ε*_*Total*_; **E** and **F)** as functions of afterload for representative **(A, C and E)** STZ (filled circles) and SHAM (open circles) working hearts, and for the averages of *n* = 16 STZ or *n* = 17 SHAM working hearts **(B, D and F)**. The variability of the relations in B, D and F is quantified in Figures 5 and 6 and Tables 2 and 3.

**Figure 5:**
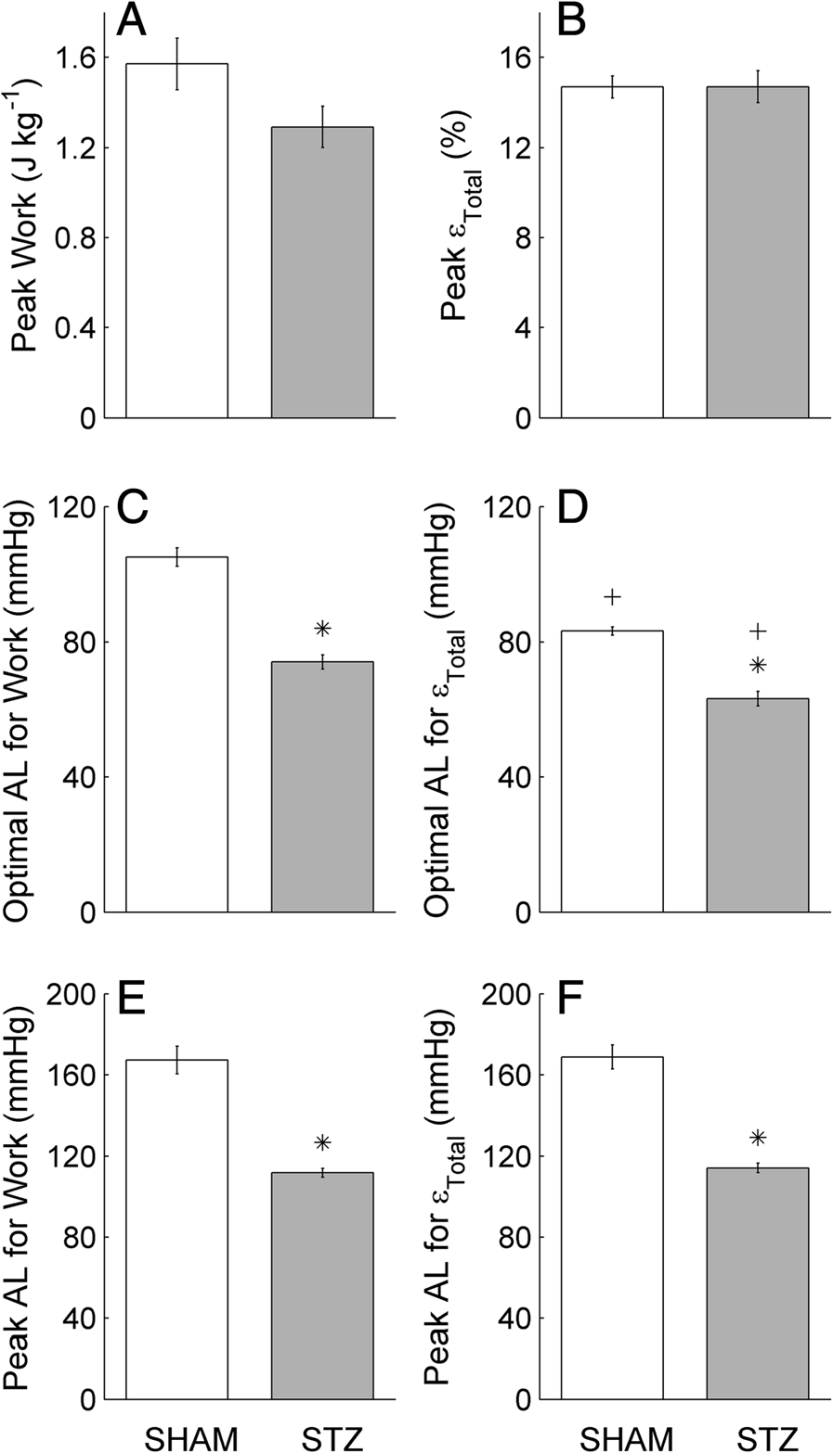
**Peak work, peak total efficiency, optimal afterloads and peak afterloads.** Quantification of the average relations shown in Figure 4B and F: peak work **(A)**, peak total efficiency (*ε*_*Total*_; **B**), the optimal afterload (AL) for work **(C)** and for *ε*_*Total*_**(D)** and the peak afterload for work **(E)** and for *ε*_*Total*_**(F)**. Values represent the mean ± SE of *n* = 17 SHAM hearts and *n* = 16 STZ hearts. *P < 0.05, STZ versus SHAM; ^+^*P* < 0.05, optimal AF for work versus that for *ε*_*Total*_.

Work, total oxygen consumption (*VO*_*2*_) and total efficiency (*ε*_*Total*_: Equation 1) were plotted as functions of afterload, as illustrated in Figure 4, where panels A, C and E show data from a representative heart from both STZ and SHAM groups, and panels B, D and F show the average relations. In all cases, the relations between these three variables and afterload were shifted to lower afterloads in diabetic hearts (quantified in Figure 5), a collateral consequence of the reduced ability of the diabetic hearts to pump at high afterload. Several features of the relations are noteworthy. (i) Consistent with data from isolated cardiac muscle, work and *ε*_*Total*_ of the isolated working heart were both complex functions of afterload, which peak at the mid-range of afterload. (ii) Neither work nor *ε*_*Total*_ were zero at the highest afterload that the heart could pump. This is because at the highest afterload, coronary flow rate was high despite near-zero aortic flow (see Figure 3), and work was calculated using the sum of aortic and coronary flow rates. (iii) Because of the previous two features, the data allowed us to extrapolate to peak afterload at the extreme positive value on the abscissa where work (or *ε*_*Total*_) was zero. (iv) Lastly, the afterloads at which the peak work was attained were not comparable to the afterloads at which the peak *ε*_*Total*_ was reached. For example, for SHAM hearts, peak work occurred at around 110 mmHg, whereas peak *ε*_*Total*_ appeared at around 80 mmHg.

**Figure 6:**
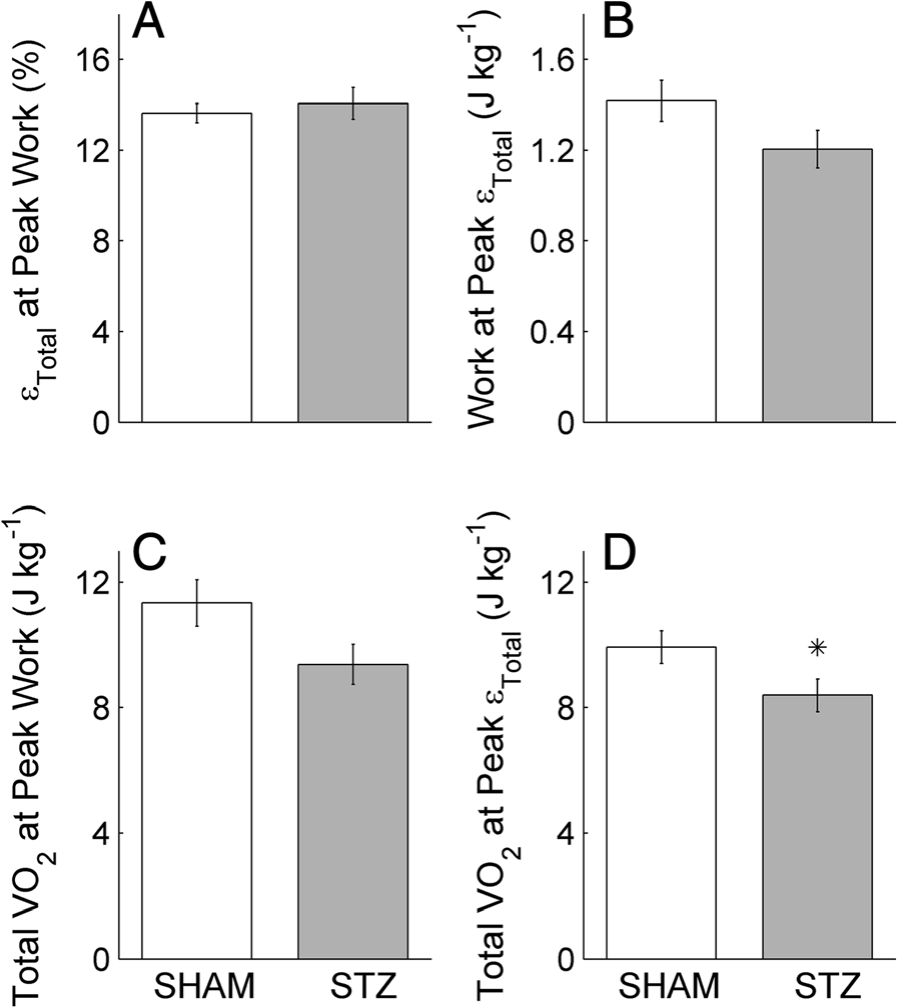
**Work and total oxygen consumption (*VO***_***2***_**) at peak total efficiency (*ε***_***Total***_**),*ε***_***Total***_**and total*VO***_***2***_**at peak work.** Further quantification of the average relations shown in Figure 4B, D and F: ***ε***_***Total***_ at peak work **(A)**, work at peak *ε*_*Total*_**(B)** and total VO_2_ at peak work **(C)** and at peak *ε*_*Total*_**(D)**. Values represent the mean ± SE of *n* = 17 SHAM hearts and *n* = 16 STZ hearts. *P < 0.05, STZ versus SHAM. In **B** and **C**, the *P* values are 0.093 and 0.056, respectively.

Given these four interesting features of the relations, we quantified four distinct variables (illustrated in Figure 1), namely: (i) peak values (i.e., peak work and peak *ε*_*Total*_); (ii) optimal afterloads (i.e., the afterloads at which peak work and peak *ε*_*Total*_ occurs); (iii) peak afterloads (i.e., the maximum, extrapolated, values of afterload); and (iv) values at the peak of other variables (i.e., values of work and total *VO*_*2*_ at the optimal afterloads of *ε*_*Total*_*,* and values of *ε*_*Total*_ and total *VO*_*2*_ at the optimal afterloads of work). Quantification of the first three variables is reported in Figure 5, whereas those for the fourth variable are shown in Figure 6.

### Peak work and peak total efficiency, optimal afterload and peak afterload

Figure 5 compares the peak work, the peak *ε*_*Total*_, the optimal afterload and the peak afterload for work and for *ε*_*Total*_ of the average relations shown in Figure 3B and F. Mean peak work (A) did not differ between STZ and SHAM hearts (*P* = 0.068). Mean peak *ε*_*Total*_ was also not different between STZ and SHAM hearts, 14.7 ± 0.7% versus 14.7 ± 0.5% (B). However, the mean optimal afterload for work (C) and that for *ε*_*Total*_ (D) were lower in the STZ hearts. The mean peak afterload for work (E) and that for *ε*_*Total*_ (F) were also lower in the STZ hearts. In addition, we also compared the mean optimal afterload and the mean peak afterload for work with that for *ε*_*Total*_ (comparing panels C and D). For both the STZ and SHAM hearts, the mean optimal afterload for work was greater than that for *ε*_*Total*_*,* but the mean peak afterload for work and for *ε*_*Total*_ were not different (comparing panels E and F).

### Work and total oxygen consumption (*VO*_*2*_) at peak total efficiency (*ε*_*Total*_), *ε*_*Total*_ and total *VO*_*2*_ at peak work

Since the optimal afterload for work (i.e., afterload at which work is at its peak) did not coincide with that for *ε*_*Total*_ (comparing Figure 5C and D), peak work did not result in peak *ε*_*Total*_*.* Hence, we calculated the work, along with the total *VO*_*2*_, at the optimal afterload for *ε*_*Total*_*.* Likewise, we calculated *ε*_*Total*_ and total *VO*_*2*_ at peak work. Note that peak *ε*_*Total*_ (Figure 5B) is the ratio of the work values in Figure 6B to the total *VO*_*2*_ values in Figure 6D. At peak *ε*_*Total*_, work did not differ between the STZ and SHAM hearts (Figure 6B), but total *VO*_*2*_ was lower for the STZ hearts (Figure 6D). At peak work, both*ε*_*Total*_ and total *VO*_*2*_ did not differ between the two groups (Figure 6A and C, respectively).

### Mechano-energetics values at afterload 70 mmHg

We measured the basal rate of *VO*_*2*_ of each heart at afterload 70 mmHg. There was no difference in the mean basal rate of *VO*_*2*_ between STZ and SHAM (Table 2). At this afterload, mean aortic flow rate and mean coronary flow rate were not different between the two groups. Therefore, mean work, total *VO*_*2*_ and total *ε*_*Total*_ were also not different between the two groups. In fact, at this afterload, the value of these variables of the STZ hearts (when plotted as functions of afterload) intersected those of the SHAM hearts (Figure 4). Given the null differences in values of these four variables between the two groups, mechanical efficiency at 70 mmHg (calculated using these four variables; Equation 2) did not differ between the STZ and SHAM hearts.

**Table 2:** Mechano-energetics values at afterload 70 mmHg

**Variable**	**SHAM**	**STZ**
Basal rate of VO_2_(W kg^-1^)	12.1 ± 1.07	11.5 ± 0.87
Aortic flow rate (mL g^-1^ min^-1^)	26.7 ± 1.6	26.1 ± 2.3
Coronary flow rate (mL g^-1^ min^-1^)	5.01 ± 0.29	5.74 ± 0.43
Work (J kg^-1^)	1.22 ± 0.07	1.24 ± 0.09
Total Vo_2_ (J kg^-1^)	8.87 ± 0.43	9.04 ± 0.59
Total efficiency (%)	14.2 ± 0.48	14.1 ± 0.77
Mechanical efficiency (%)	21.4 ± 0.97	20.9 ± 1.26

Values are means ± SE. No statistically significant differences in mean values between SHAM and STZ.

### Mechano-energetics values at afterload 90 mmHg

We chose to report the values of the mechano-energetics variables at 90 mmHg since at this afterload the STZ hearts were in the vicinity of their limit to pump (Figures 3 and 4). In contrast to the null results at afterload 70 mmHg (Table 2), results at 90 mmHg are striking. As shown in Table 3, the mean aortic flow rate of the STZ hearts was lower, resulting in lower average work performance. This result, in turn, was responsible for the lower value of *ε*_*Total*_ since there was no difference in the mean value of total *VO*_*2*_ between the two groups.

**Table 3:** Mechano-energetics values at afterload 90 mmHg

**Variable**	**SHAM**	**STZ**
Aortic flow rate (mL g^-1^ min^-1^)	24.4 ± 1.5	14.2 ± 2.9*
Coronary flow rate (mL g^-1^ min^-1^)	6.05 ± 0.39	7.16 ± 0.65
Work (J kg^-1^)	1.48 ± 0.09	1.06 ± 0.11*
Total Vo_2_ (J kg^-1^)	10.4 ± 0.51	10.0 ± 0.66
Total efficiency (%)	14.5 ± 0.49	10.4 ± 0.88*

Values are means ± SE. Asterisks indicates significant differences in mean values between SHAM and STZ: *P < 0.05.

## Discussion

To our knowledge, this study presents the first comprehensive examination of the mechano-energetics of the diabetic heart. We have extended the practice (common in the literature) of reporting only a single value of myocardial efficiency of the diabetic heart by reporting its behaviour as a function of afterload. Efficiency is a complex function of afterload, with values ranging from zero to a peak (which occurs at somewhere in the mid-value of afterload). It is thus incomplete, and misleading, to report only a single value. We show that the efficiency-afterload relation allows reconciliation of the conflicting conclusions in the literature regarding the effect of diabetes on myocardial efficiency.

### Achievement of the diabetic state

A single injection of STZ at age 6-7 weeks, clearly rendered the rats diabetic 7-8 weeks later. This is demonstrated by the 4.4-fold higher concentration of blood glucose, the 30% lower body mass and the 23% lower heart mass but 11% higher ratio of heart mass to body mass (Table 1). The STZ rats developed LV hypertrophy, as indicated by a greater average LV wall thickness relative to heart mass (Table 1), consistent with the finding of MacDonald et al. [32]. Compared to their SHAM controls, the STZ rats had a greater average lung mass per body mass, consistent with the findings of Ofulue et al. [33]. They also had lower average intrinsic heart rates, as measured *in vivo* and *in vitro* (at 32°C). Bradycardia has been a consistent finding in STZ-diabetic hearts [19,20,22-25,32,34-36]. We obviated any influence of bradycardia on the mechano-energetics variables by pacing the excised hearts at 4 Hz (i.e., above the intrinsic heart rates at 32°C).

### Adequacy of oxygen supply to the isolated working-heart

The use of a crystalloid perfusate demands examination of the adequacy of oxygen supply. We exploited the inverse relationship between temperature and oxygen solubility–lowering the temperature of the perfusate to 32°C, which, in turn, allowed us to reduce the pacing frequency to 4 Hz. Thus we simultaneously increased oxygen supply and reduced oxygen demand, both of which manoeuvres enhance tissue oxygenation. As seen in Figure 2, the average coronary venous PO_2_ was 25 mmHg at the lowest workload examined (40 mmHg afterload). This value can be put in perspective with reference to Tune et al. [37] and the comprehensive review of Zong et al. [38], who reported that resting PO_2_ values from the right and left coronary veins of dogs are, respectively, 30 mmHg and 20 mmHg. It is unlikely that our observed values indicate hypoxic conditions, given the substantial coronary reserve revealed by the progressive increase of venous PO_2_ values with afterload (consistent with the *in situ* canine data of Yonekura et al. [39]). Note that the average highest value of venous PO_2_ (which occurred at the maximal afterload) was 90 mmHg for diabetic hearts and 140 mmHg for control hearts. PO_2_ values above 100 mmHg exceed those associated with normoxia *in vivo*. Also observed in Figure 2 is a hint of autoregulation in the mid-range of afterloads and the probable appearance of Gregg’s Phenomenon at higher rates of work demand. These considerations give us confidence that the supply of oxygen to the isolated hearts was adequate and allow us to focus on the effects of diabetes on cardiac mechano-energetics.

### Blood pressure in vivo

Given the development of bradycardia and LV hypertrophy in the diabetic rats, it is somewhat surprising that no difference of either systolic or diastolic blood pressure was observed *in vivo* (Table 1). Systolic blood pressure, as well as mean arterial pressure and pulse pressure, have been shown to be higher and to increase with the duration of diabetes in Type 1 diabetic patients [40,41]. Elevated blood pressure has been used as an index of arterial stiffening in diabetic patients [40,41]. Arterial stiffness is known to be higher in diabetic human tissue *post-mortem*[42] as well as in the arteries [43-45] and myocytes [44] of diabetic patients. These effects are commonly attributed to increased fibrosis [44], deposition of advanced glycation end-products [44,45], increased oxidative stress [45] or low-grade inflammation [43]. The absence of any difference of blood pressure indices between STZ and SHAM rat hearts probably reflects the difference between acute (present study) and chronic diabetes (human patients).

### Aortic flow, coronary flow and maximal afterload in vitro

The effect of STZ-induced diabetes was primarily manifested on the rate of aortic flow (Figure 3B). The extent of decrease of the aortic flow with increasing afterload was greater for the STZ hearts, resulting in negligible flow at an average afterload of 105 mmHg (Figure 3). In contrast, the SHAM hearts did not achieve zero aortic flow until challenged by an average afterload of 150 mmHg. This demonstrates that the LV of the STZ heart fails to generate as high a pressure as the normal heart *in vitro*. This finding is consistent with the reduced peak developed LV pressure of aortic-occluded diabetic hearts [25] and the reduced peak LV systolic pressure reported by others [4,9,21-25].

However, there are also studies showing no effect of STZ on LV systolic pressure [1,2,19,20]. Nevertheless, it is striking that the clear difference in maximum LV pressure development between STZ and SHAM hearts *in vitro* (Figure 3) stands in contrast to the absence of any differences among systolic, mean or diastolic pressures measured *in vivo* (Table 1). The literature on these discrepant results once again lacks consensus. Whereas it is not uncommon to read that STZ-diabetic cardiomyopathy in rats lowers mean arterial pressure significantly [46-49], most groups have reported no difference [19,20,23,34,35,49-51], while some have even reported higher mean values [52,53]. Clearly, any observed response of either mean arterial pressure or LV pressure can be accommodated. In fact, both Radovits et al. [23] and Cheng et al. [35] reported lower *in vivo* LV systolic pressure with no change of mean arterial pressure in the STZ-diabetic rats. Our results, showing reduced maximal afterload (Figure 3) and similar mean arterial pressure (Table 1), are in accord.

At the maximum afterload (when the aortic flow rate is near zero), the coronary flow rate is maximal. STZ-treatment has negligible effect on the maximum rate of coronary flow (8-10 mL min^-1^ g^-1^), in accord with the finding of Joffe et al. [24] who showed no effect of STZ on coronary perfusion pressure of the *in vitro* heart. This suggests that autoregulation of the heart, which is responsible for the relatively constant coronary flow within an afterload region between around 60 mmHg to 100 mmHg (Figure 3), is unaffected by STZ treatment. At the maximum afterload, since the coronary flow rate is maximal, the derived work output of the heart is small but non-zero (since aortic flow rate is near zero; Figure 4A). In consequence, the calculated total efficiency (Equation 1) is a small value (Figure 4E). This has prompted us to extrapolate the curves fitted to the work-afterload and efficiency-afterload data to predict the peak value of afterload. These extrapolated values approximate the systolic pressure of an isovolumic contraction. Since the predicted maximum afterload of the STZ hearts is lower (105 mmHg versus 150 mmHg; Figure 3), the predicted peak afterload (or isovolumic pressure) of the STZ hearts is consistently lower (114 mmHg versus 169 mmHg; Figure 5E and F). Reassuringly, the predicted peak afterload does not differ in value whether it is extrapolated from the work-afterload curve (Figure 5E) or from the efficiency-afterload curve (Figure 5F).

### Peak values of work and of total efficiency in vitro

These two peak values occur at neither the maximum afterload nor the predicted peak afterload. Instead, they occur at their optimal afterloads, as shown in Figure 4 and quantified in Figure 5C and D. Given that STZ reduces the maximum afterload and the predicted peak afterload, the optimal afterload is necessarily reduced. Despite this effect, the peak value of total efficiency (14-15%) is unaffected by STZ (Figures 4F and 5B). These values of total efficiency fall within the range of the literature values reported for the normal rat heart [54].

Note that the average efficiency-afterload curve of the STZ hearts intersects that of the SHAM hearts at an afterload of approximately 70 mmHg (Figure 4F). At this afterload, diabetes has negligible effects (Table 2) on either aortic or coronary flow rates, work output, total oxygen consumption, total efficiency, or basal rate of oxygen consumption. Correction for the basal rate of oxygen consumption (Equation 2) reveals that the mechanical efficiency, at least at afterload 70 mmHg, is unaffected by STZ.

### Optimisation of the heart for mechano-energetics

As is also evident in Figures 4 and 5, the optimal afterload for peak work is different from that for peak total efficiency. The former occurs at a greater value of afterload than the latter, and this is independent of diabetic status. This result, in the intact whole-heart, is consistent with the results obtained in isolated one-dimensional cardiac preparations, such as papillary muscles [10-16] and trabeculae carneae [17,18].

We can calculate the value of work at which total efficiency is at its peak, and *vice versa* (Figure 5). But since the optimal afterload for peak work is greater than that for peak total efficiency, this has raised a question as to whether the STZ and SHAM hearts, *in vivo*, operate at some high afterload (90 mmHg, for example) or at the afterload that optimises either work or total efficiency. If they operate at their optimal afterloads for peak work, then they have the same total efficiency. If they operate at their optimal afterloads for peak total efficiency, then they again have the same total efficiency. But if they both operate at high afterload, then the STZ heart would have a relatively lower total efficiency (Table 3). The literature on the concept of ‘optimisation’ (the matching of either peak work or peak efficiency to arterial load) is inconclusive [55-62], but, as pointed out by Elzinga and Westerhof [63] and by de Tombe et al. [64], it scarcely matters. The afterloads at which these two maxima occur do not differ greatly, i.e., both the work and efficiency are greater than 90% of their respective optima. Our results, for both the STZ and SHAM hearts, are in accord. Thus, from the literature, we think that the heart operates within the range of optimal afterloads for peak work and for peak total efficiency. Our results, on isolated hearts, show that the STZ-diabetic heart operates optimally at afterloads 60-70 mmHg, lower than those for the healthy heart (80-100 mmHg). Despite operating at a lower afterload, the STZ-diabetic heart pumps as efficiently as the healthy heart (14.7%). We thus infer that the diabetic heart, under normal conditions, pumps as efficiently as the healthy heart, but if it is challenged with any intervention that requires it to pump at high afterloads, then its efficiency will be lower than that of the healthy heart.

### Reporting a single value of efficiency

Because of the complex dependencies of efficiency on afterload, it is unwise to perform experiments at only a single afterload, and to report a single value of efficiency, unless one has *a priori* knowledge of the relation between efficiency and afterload. If we had performed only a single experiment at a high afterload (for example, 90 mmHg), to examine the effect of diabetes, then we would have reported that the diabetic heart has a lower value of efficiency. We consider it necessary that experiments be performed at a range of afterloads since, by doing so, the optimal afterload at which the heart operates can be ascertained. If only a single value of efficiency is reported, then it is essential to report the afterload at which that value was obtained. In fact, this was done by Penpargkul et al. [9] who reported that they had performed all measurements at a fixed afterload of approximately 60 mmHg. It was their serendipitous choice of this value that allowed them to find no effect of diabetes upon myocardial work output, oxygen consumption or total efficiency – a result in accord with our findings reported in Figure 4.

## Conclusions

Streptozotocin-induced diabetes limits the ability of the heart to pump at high afterloads. A collateral consequence is a left-shift of the myocardial efficiency-afterload curve, resulting in reduced efficiency of the diabetic heart at high afterloads. However, the peak efficiency (which occurs at the mid-range of the efficiency-afterload curve) of the heart is unaffected by diabetes. These findings reconcile the conflicting reports in the literature regarding the effect of diabetes on myocardial efficiency.

## Abbreviations

STZ: Streptozotocin; RV: Right-ventricle/ventricular; LV: Left-ventricle/ventricular; PO2: Partial pressure of oxygen; VO2: Oxygen consumption; εTotal: Total efficiency.

## Competing interests

The authors declare that they have no competing interests.

## Authors’ contributions

J-CH conceived and designed the study, performed experiments and was responsible for acquisition, interpretation and statistical analysis of data, drafting the manuscript and preparing the figures. DL participated in study concept and design and contributed to acquisition, interpretation and statistical analysis of data and drafting of the manuscript. SY and AT contributed to acquisition and participated in interpretation of data. CB performed the *in vivo* measurement and participated in interpretation of data. KM contributed to interpretation of data and writing of the manuscript. All authors participated in critical revision and approved the final version of the manuscript.
